# Leaf disease image retrieval with object detection and deep metric learning

**DOI:** 10.3389/fpls.2022.963302

**Published:** 2022-09-13

**Authors:** Yingshu Peng, Yi Wang

**Affiliations:** ^1^Lushan Botanical Garden, Chinese Academy of Sciences, Jiujiang, China; ^2^College of Forestry, Nanjing Forestry University, Nanjing, China; ^3^Jiangsu Wiscom Technology Co. Ltd., Nanjing, China

**Keywords:** leaf disease recognition, image retrieval algorithm, deep metric learning, object detection, convolutional neural networks

## Abstract

Rapid identification of plant diseases is essential for effective mitigation and control of their influence on plants. For plant disease automatic identification, classification of plant leaf images based on deep learning algorithms is currently the most accurate and popular method. Existing methods rely on the collection of large amounts of image annotation data and cannot flexibly adjust recognition categories, whereas we develop a new image retrieval system for automated detection, localization, and identification of individual leaf disease in an open setting, namely, where newly added disease types can be identified without retraining. In this paper, we first optimize the YOLOv5 algorithm, enhancing recognition ability in small objects, which helps to extract leaf objects more accurately; secondly, integrating classification recognition with metric learning, jointly learning categorizing images and similarity measurements, thus, capitalizing on prediction ability of available image classification models; and finally, constructing an efficient and nimble image retrieval system to quickly determine leaf disease type. We demonstrate detailed experimental results on three publicly available leaf disease datasets and prove the effectiveness of our system. This work lays the groundwork for promoting disease surveillance of plants applicable to intelligent agriculture and to crop research such as nutrition diagnosis, health status surveillance, and more.

## Introduction

As one of the hottest topics in intelligent agriculture, plant disease detection has received unprecedented attention recently. The solution to this task is crucial to meet multifarious challenges in agriculture such as sustainable development, productive forces, and environmental implications. In actual production, this task is normally completed by artificial classification. But this complex task generally requires seasoned experts who are frequently limitedly available. Various automatic classification algorithms relying on convolutional neural networks (CNNs) have been constructed to solve these difficulties. As the most representative algorithm in deep learning, CNN has achieved great success in computer vision, information retrieval, natural language processing, and many other fields. In recent years, CNN is becoming more and more popular for diagnosing plant leaf diseases. If we look at the literature, we can recognize the effectiveness of existing deep learning methods for diagnosing plant diseases (Abade et al., 2021; Kundu et al., 2021). For existing methods, they can be further subdivided into classification methods, objective detection methods, and segmentation methods according to the network structure used (Dhaka et al., 2021; Liu and Wang, 2021).

As for classification methods, because of CNN's powerful feature extraction ability, the adoption of CNN-based models has become the most commonly used pattern in plant disease identification. Most of them utilized classical CNN models for transfer learning or feature extraction (Li et al., 2021). There are also some studies that have designed network structures based on practical problems (Ahila Priyadharshini et al., 2019; Sunil et al., 2022). A lot of scientific research also employed segmentation networks or detection networks for plant disease identification. For example, YOLOv5 was used for detecting the bacterial spot disease in the bell pepper plant, which can detect even a small spot of disease with considerable speed and accuracy (Mathew and Mahesh, 2022). Chouhan et al. (2021) proposed a neural network model with superpixel clustering for segmentation and achieved 98.57% detection accuracy. However, no matter which method is used, most of these methods classify plant disease images based on CNN network to diagnose and quantify plant diseases.

Although good progress has been made over the past years, these image classification-based methods still suffer from some problems that limit the practical applications of plant leaf disease recognition algorithms based on image classification networks. These problems are mainly manifested in three aspects:

1. The recognition category cannot be adjusted flexibly. In practical applications, the categories of plant leaf diseases usually change frequently in different scenarios, and the number of categories that can be predicted by classification algorithms including image classification networks is fixed. This severely limits practical applications of current approaches, as each time the network needs to be modified and retrained when adding or reducing recognition categories (Guo et al., 2019).
2. The number of recognition categories is restricted. In various detection scenarios, the categories of leaf disease are huge and diverse, and the number of parameters and calculations of the image classification network will many fold grow as add number of recognition categories, which requires powerful computer capability and data storage, but this severely hinders model training and deployment on hardware devices with limited performance. Therefore, the recognition algorithm based on image classification networks is difficult to apply to leaf disease recognition applications with a large number of categories.
3. Over-reliance on labeled data. Numerous current image classification for visual recognition tasks commonly relies on large amounts of labeled training data to achieve high performance (Liu et al., 2020a). Data collection and labeling of leaf diseases are commonly challenged, either because of high cost or lack of appropriate expertise. Under the condition of limited training samples, it is problematic for image classification models to obtain preferable identification results in leaf disease diagnosis.

The study focuses on the issues listed above. Accordingly, we put forward an image retrieval system based on object detection and deep metric learning to identify plant leaf diseases. Our system can identify leaf diseases quickly and accurately and overcome the shortcomings of leaf disease identification methods based on image classification, which has high theoretical research and engineering application value.

Simply put, the primary advantages and contributions of our proposed retrieval system are as follows:

1. A detection algorithm was proposed based on object detection algorithm and deep metric learning, which can locate and identify plant leaves and disease types that seldom appear or never appear before in the training dataset. The training data required is considerably reduced and gets a near image classification algorithm performance.
2. We designed a complete but simple image retrieval system structure using the reconfigurable and module method, without employing any complicated projects (such as network compression, vision transformer), and gradually analyzed the impact of different hyper-parametric techniques and models on performance with a large number of experiments. Recognition categories and each module of our system are allowed to adjust quickly and freely within actual task requirements.
3. Using the image retrieval system to improve the versatility of the algorithm. The leaf disease detection model trained on a large-scale dataset can be directly applied to various practical leaf disease identification tasks without retraining or fine-tuning. This can be confirmed by the application and development of technologies such as person reidentification (Zhong et al., 2017) and face recognition (Liu et al., 2018). Thus, the modules of our algorithm are separated, allowing task-by-task optimization to enhance system performance.

Three different datasets were considered in the test: PlantVillage dataset (Hughes and Salathe, 2016), coffee leaf dataset (Esgario et al., 2020), and citrus leaves dataset (Wu et al., 2007). By evaluating our methods in a large number of experiments with plant disease images, we demonstrate that our novel image retrieval framework can address plant disease detection in small samples and complex environments from a new perspective by combining object detection and metric learning technology.

## Related work

### Metric learning

Metric learning focuses on automatically extracting a robust metric from images to precisely determine similarity or distance between different images. The common methods for metric learning are roughly divided into two types: Siamese networks coupled with contrastive loss (Simo-Serra et al., 2015) and triplet networks coupled with triplet loss (Wang et al., 2014). Since it has been proved that triplet loss outperforms contrastive loss (Liu et al., 2020b) in most cases, we adopted the former in this paper.

**Figure 1:**
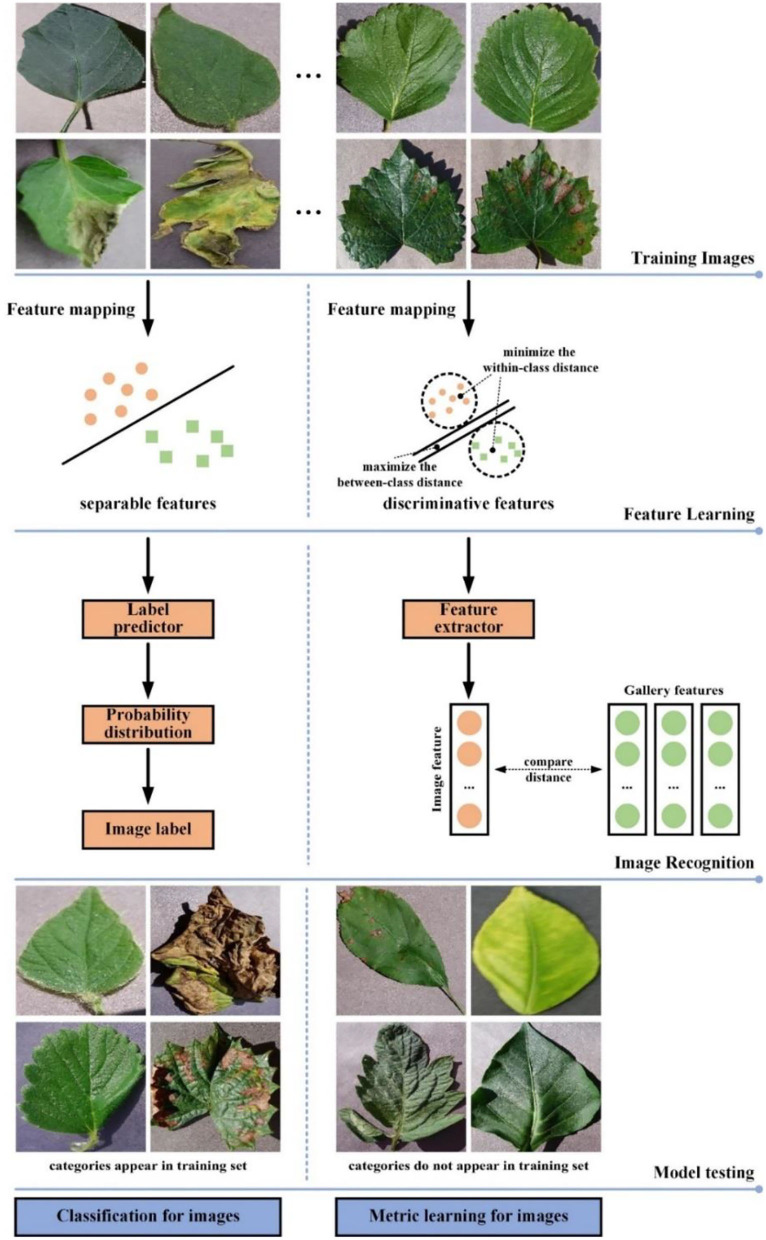
Comparison of image classification and image retrieval in leaf disease recognition.

The difference between image classification approaches and metric learning approaches is shown in Figure 1. The goals of classifier training and metric learning are different. The CNN network trained using classification loss makes the classification task easier with learning separable features from images. To obtain correct recognition result, metric learning selects the discriminative features so that make the distance of the same class images in the feature space as close as possible, while that of different classes images further wherever possible. Therefore, metric learning is more appropriate for small samples or multi-category classification scenarios than classification learning. However, unlike classification loss, metric learning loss cannot constrain each individual sample, which will lead to instability of metric learning loss during model training (Ma et al., 2021). To alleviate this problem, this paper would perform similarity measurements and categorize images through integrated metric learning with classification prediction.

### Image retrieval

**Figure 2:**
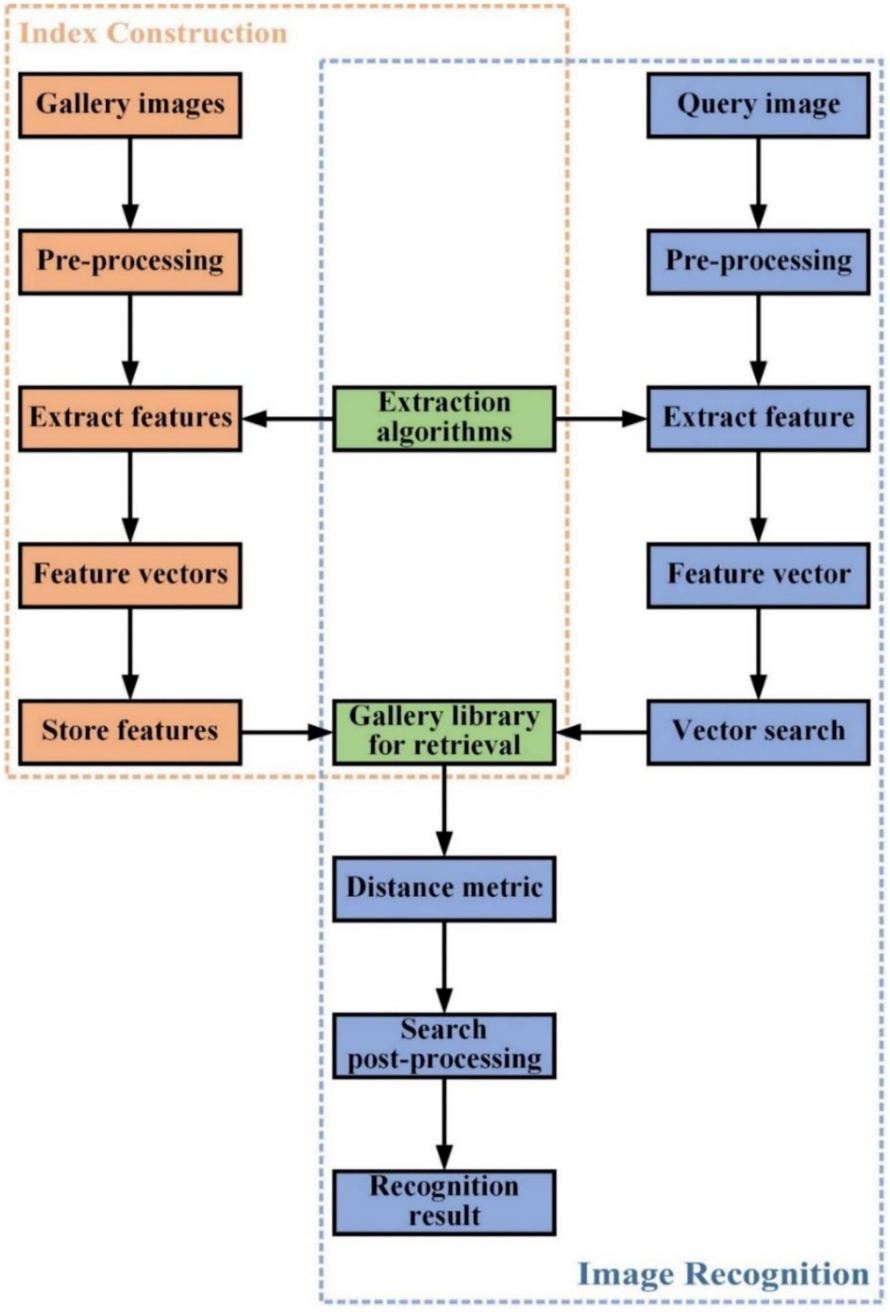
Workflow of image retrieval.

Image retrieval is a well-explored problem in computer vision research. Its goal is to find one or more images containing the same target or scene as the query image from a collection of images (Yang et al., 2019). Unlike image classification tasks, image retrieval solves a problem where the testing categories are generally different from those used in training (Jiang et al., 2021). There are two paradigms for image searching: content-based image retrieval and text-based image retrieval (Nag Chowdhury et al., 2018). Content-based image retrieval utilizes image search techniques that combine vision features to answer queries. Text-based image retrieval manages data and seeks an image that best matches the query text from a set of images through traditional database techniques. The image retrieval mentioned in this paper refers to content-based image retrieval since it directly uses vision features extracted from images for retrieval. Figure 2 shows the general process of image retrieval.

The overall process of image retrieval is: firstly, the images are represented in a suitable feature vector, and get image features from existing images to generate retrieval indexes for building a gallery library. Secondly, a search method is performed on this image feature vector using Euclidean or Cosine distances to find similar images in the gallery library, and finally, some post-processing techniques can be used to fine-tune the retrieval results and determine information such as the category of the image being recognized.

Feature extraction generalizes images into high-dimensional feature vectors, the quality of which plays a pivotal role in determining retrieval performance. Here, we employ deep metric learning to extract abstraction features from image data and then measure similarities among images.

According to sample data specific purpose in image retrieval, it can be divided into three parts:

1. Training dataset: Used to train the model so that it can learn the image information of the collection.
2. Gallery dataset: Used to provide gallery data for image retrieval tasks. The gallery dataset can be the same as the training set or the test set, or different.
3. Query dataset (test dataset): Used to test the goodness of the model.

### Closed-set identification and open-set identification

Image identification is an important task in computer vision, which can commonly be categorized as closed-set identification and open-set identification according to whether classes in the test set appear in the training set (Bendale and Boult, 2016). For closed-set identification, all classes in the test set are restricted to the classes seen before in the training set. In the case of open-set identification, classes of the training set disjoint from those of the test set. The open-set identification task is a more common situation, with the closed-set identification being its particular case; therefore, the difficulty of the former is typically considerably greater than that of the latter.

The leaf disease detection method based on image classification can only solve closed-set image identification, while our method based on image retrieval proposed in this paper can both perform closed-set identification and open-set identification. We will test the efficiency of our work by performing closed-set identification and open-set identification on datasets.

## Methods

In this section, we will systematically describe our method and emphasize its main advantages.

### Overall framework

**Figure 3:**
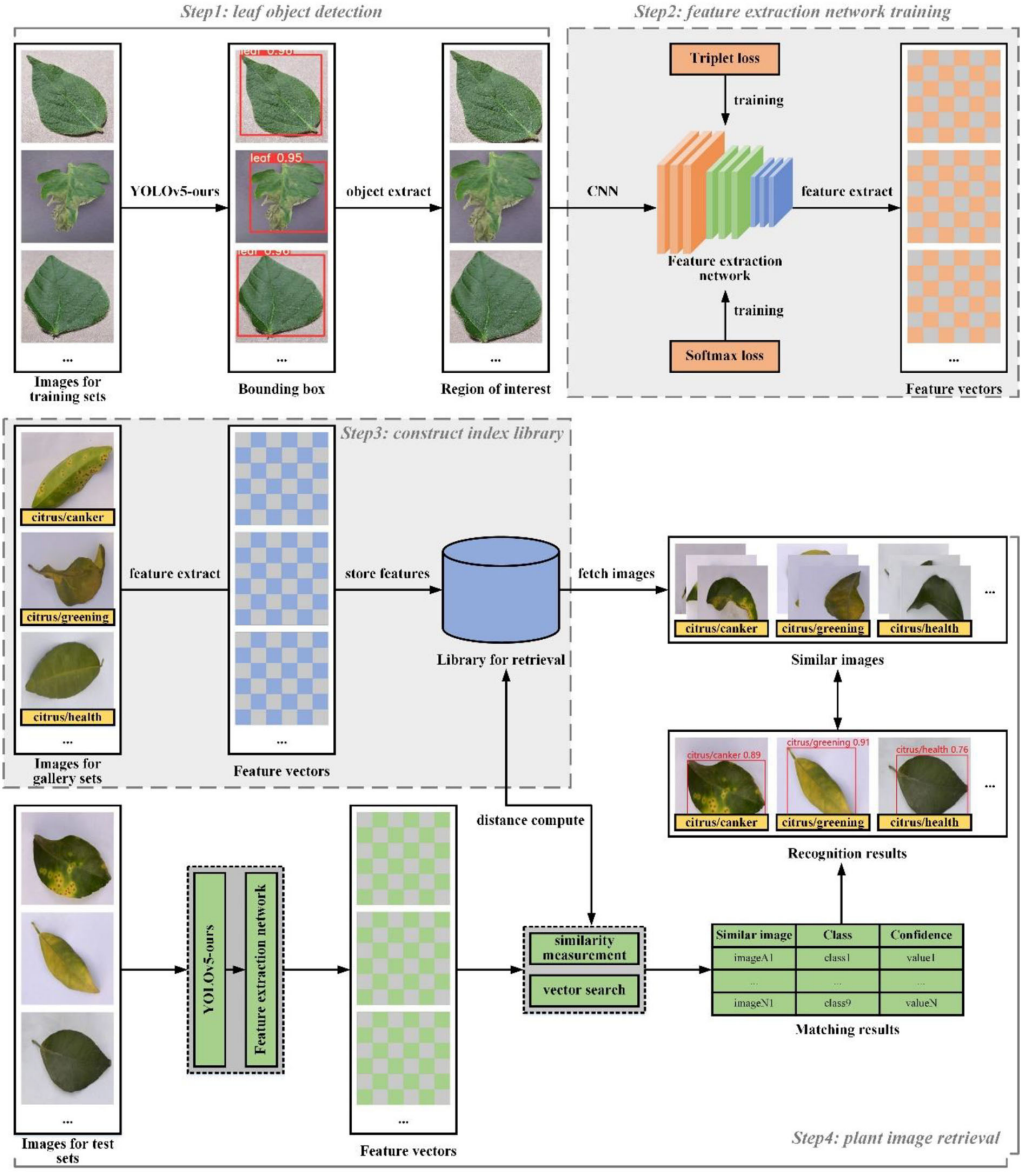
The framework of our image retrieval system.

As demonstrated in Figure 3, the framework of our image retrieval system is given.

The framework is mainly composed of four parts, namely, leaf object detection (Step 1), feature extraction network training (Step 2), construct index library build (Step 3), and plant image retrieval (Step 4). These modules are briefly described below. Technical details will be described in the rest of this section.

Leaf object detection algorithm is responsible for predicting the bounding box location for each leaf in an image. The feature extractor is used to generate a feature vector for each leaf region. The feature extraction algorithm and the object detection algorithm are independent of each other by training completely different datasets, so they can be carried out separately during the training process.

In practical application of leaf disease image retrieval system, the number of categories that need to be recognized is commonly very large or unknown. For fast retrieval, the nearest neighbor search is then used on the search between query vectors and index vectors and is performed to obtain matching prediction categories. Therefore, before image retrieval system deployment, we will extract given image features into the form of feature vectors and build an index library according to the corresponding categories. For a new leaf disease category, we only need to put images of this category into the retrieval database, so that our retrieval system can identify this new leaf disease without further retraining.

### Leaf object detection

The first stage in our image retrieval framework is the automatic detection and localization of leaves within images. That is, we train a generic leaf detector such that for an input leaf image, and then get bounding box coordinates with confidence scores enclosing every leaf region within it as output. There are obvious differences in phenotypic characteristics among different varieties of plant leaves, with each individual presenting a varying texture and shape. How to accurately and timely locate leaves in a complex environment is a huge challenge for many detection algorithms. In object detection of deep learning (Song et al., 2021), YOLO series models have the best composite performance that effectively balances accuracy and speed. YOLOv5 is based on YOLOv3 (Redmon and Farhadi, 2018) and adopts the model scaling technology of EfficientDet (Tan et al., 2020) to realize dynamic adjustment of accuracy, speed, and model parameters, which has been widely used in practical tasks. Therefore, we believe that YOLOv5 is most suitable for leaf detection.

YOLOv5 was first released on GitHub 4 in May 2020 (Ultralytics/yolov5, 2021), and its version v5.0 is used in our experiments. Moreover, according to model structure and its layer channels different in set width and depth factor, several models can be chosen in YOlOv5 to meet diverse circumstances. Object detection is a very time-consuming task; so in order to reduce detection time, we adopt YOLOv5s, the lightest model among all YOLOv5 networks, as our base object detection structure.

However, YOLOv5s cannot accurately handle the detection of small leaf objects in a complex environment. As the leaf has small size and few pixel features in some images, the detection model is required to have a strong ability for small objects. In the original YOLOv5s model, the feature map of the last layer of the backbone network is too small to meet the requirements of the subsequent detection and regression. To solve this problem, we add a small object detection layer and modify or remove some layers.

**Figure 4:**
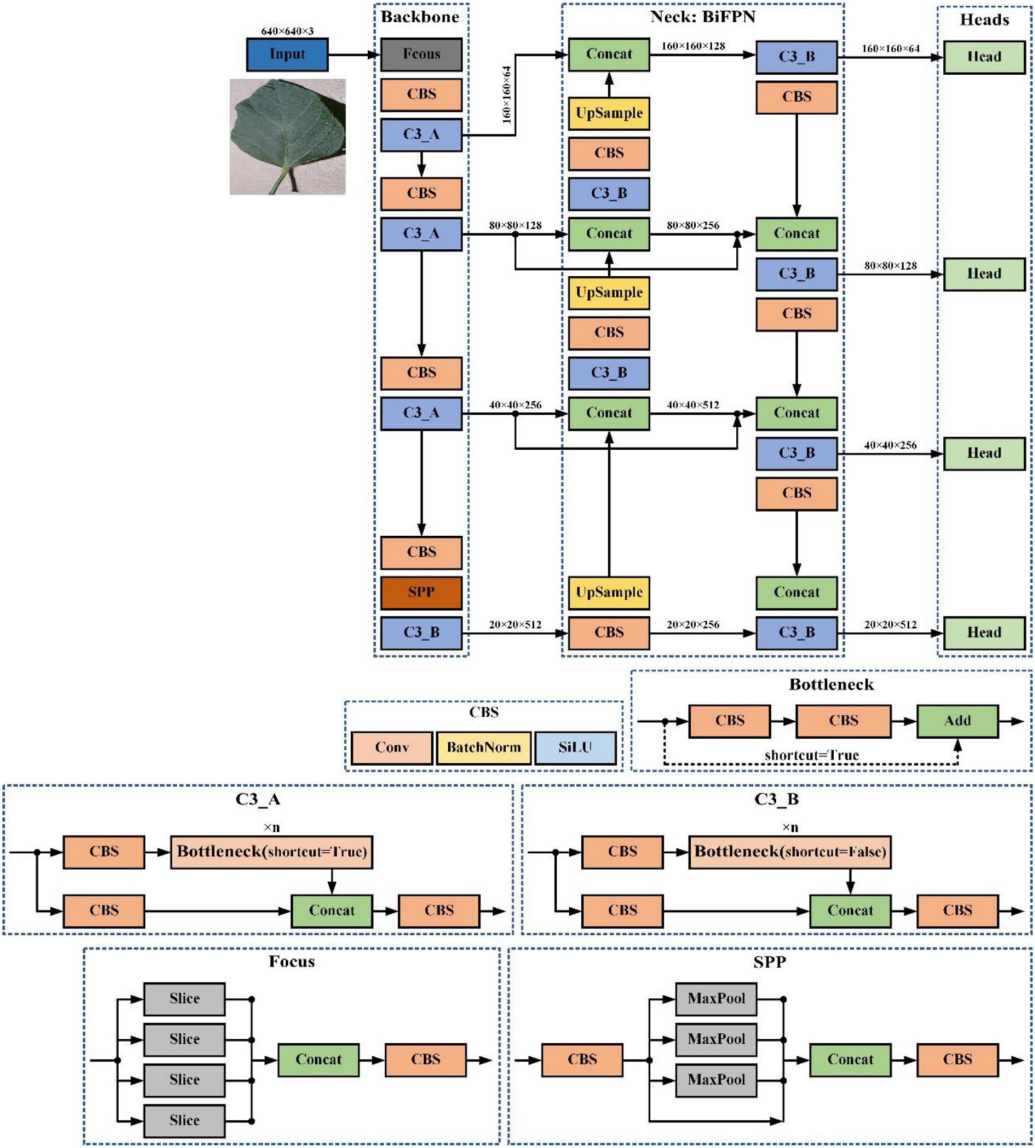
YOLOv5-our structure overview.

In detail, the acquired feature map and the feature map of the second layer in the backbone network are fused to generate a larger feature map for small object detection. Moreover, in the feature pyramid structure of YOLOv5s, we also introduce the shortcut connection used in the weighted bidirectional feature pyramid network (BiFPN) structure to better combine representations of an image at different resolutions (Tan et al., 2020). YOLOv5-ours consists of three components: a backbone network, a neck module (BiFPN), and a detection head, whose whole structure is shown in Figure 4.

In Figure 4, Input refers to network input. UpSample represents an upsampling operation, Concat denotes a concatenation operation, and Conv denotes a convolution operation. The CBS block is composed of a convolution layer, a batch normalization operator, and a SiLU activation function. The YOLOv5-our model contains two cross-stage partial structures, of which the C3_A structure is applied to the backbone of the network, while the C3_B structure is used in the neck of the network. Both C3_A and C3_B are composed of several bottleneck modules and other core modules. The only difference between them is that the bottleneck module of C3_A contains a shortcut connection, but that of C3_B does not.

1. Input: The input end of YOLOv5-ours uses the same data augment method as YOLOv4, which performs better in small object detection. YOLOv5s adds the function of adaptive anchor frame calculation. In the training process, adaptively calculate the value of the best anchor frame in different training sets.
2. Backbone: Our model aggregates and forms image features on different types of image granularity through the backbone. In addition, YOLOv5s-ours uses the Focus structure to realize the slicing operation. For example, the original 640 × 640 × 3 image is fed into the Focus module, and it finally constructs a feature map of 320 × 320 × 32. The main function of the Focus structure is to reduce floating point operations and improve the running speed of the model. The backbone further incorporates a spatial pyramid pooling (SPP) block, which performs concat operations on feature maps of different scales to allow dynamic setting of input image sizes.
3. Neck: YOLOv5-ours uses the BiFPN structure as neck to aggregate features and pass the image features to the prediction layer, strengthening detection of different scale objects. This structure enhances the bottom-up path and adds the shortcut path which improves propagation of low-level features.
4. Heads: Our model uses the same head architecture as YOLOv5s, which can handle image features, generate prediction categories, and export bounding boxes.

To generate predictions with only a high degree of confidence, the minimum confidence threshold for detection is set at 0.70. Except for the model structure, other parameters, training strategy, and loss function of our model are the same as YOLOv5s.

### Feature extraction network based on metric learning

The goal of our feature extraction network is to extract robust and discriminant semantic information from the leaf image that is mapped to a fixed dimension feature vector. This feature vector will finally be matched with the same claimed identity vector to obtain leaf disease recognition result. It can be noticed that the quality of extracted features essentially determines system retrieval accuracy. Therefore, we proposed a feature extraction network that combines metric learning and standard supervised classification prediction to heighten the model's stability and precision.

#### Network architecture design

**Figure 5:**
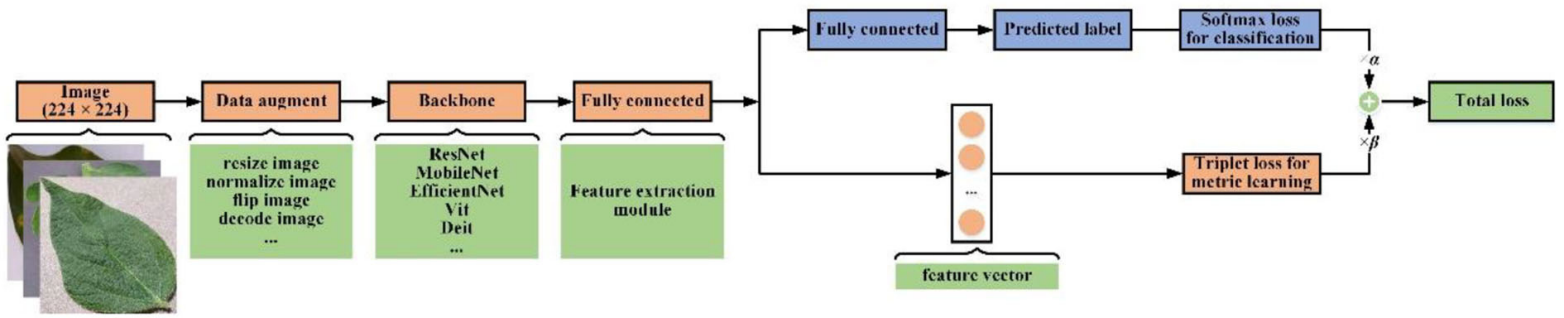
The feature extraction network structure diagram.

The structure of our feature extraction network is illustrated in Figure 5. The network mainly includes a data augment module, a backbone network, a feature extraction module, and a classification module.

Data augment module: We use standard data augmentation pipeline typically used in classification tasks, such as, randomly cropping the image to size with 224 × 224, normalizing the image pixel values, randomly flipping the image horizontally, decoding images, and so on.

Backbone network: The backbone network is a pre-trained image classification network whose classification layer is removed. This network can be any common classification CNN.

Feature extraction module: Generally, deep features extracted from most backbone networks are ultra-high dimensions, and direct use of such features will lower vector search efficiency in image retrieval and attach additional computations. So, in the feature extraction module, the fully connected layer is deployed to compress the output of the backbone network into prefixed size feature maps for our tasks. This fixed size choice was found on the existing study, and empirical values typically are 128, 256, and 512. In Section Analysis of leaf disease recognition algorithms, we will conduct experiments to test the impact of this parameter toward detection performance. The fully connected layer is adopted to convert feature maps into a single dimension feature vector, that is, the required feature vector.

Classification module: This module consists of a fully connected layer to perform leaf disease image classification. Its input dimension is consistent with the feature vector size, and the output dimension is determined according to sample categories numbers in the training set. This module is only added to make model training more stable with faster convergence. During the testing phase of image retrieval, the category prediction results have become practically insignificant. So, after training, the classification module is removed from the trained network.

#### Selection of backbone network

Feature extraction ability of our selected backbone network directly affects feature vector quality. Our proposed framework can use any standard CNN backbone or transformer network backbone in the computer vision field, such as ResNet, MobileNetV3, Vision Transformers (ViT), Data-efficient Image Transformer (DeiT). Recently, compared with the excellent CNN network (ResNet, MobileNetV3), transformer networks (VIT and DeiT) have been shown to achieve advanced results on various computer vision tasks. It seems better to choose a complex network with significant feature extraction capabilities in our tasks, such as Vision Transformers or ResNet151. However, due to the high parameter complexity of transformers and ResNet151, which results in huge training and inference costs, more training data samples are required (Khan et al., 2021). Our principle is to choose ResNet50 since it balances efficiency and accuracy. In the process of building a feature extraction network, we removed the linear classification layer of ResNet50. Therefore, when the input size of the feature extraction network is 224, the backbone network will provide a 1 × 2048-sized feature map at the output.

#### Loss function

A suitable loss function selection is a necessary way to acquire good performance in the training process of a feature extraction network. We use two types of loss functions: softmax cross-entropy loss (softmax loss) and triplet loss. Triplet loss is based on feature distances, while softmax loss is based on the output of classification module. In the training process, we combine the two loss functions and minimize both at the same time.

##### Triplet loss

**Figure 6:**
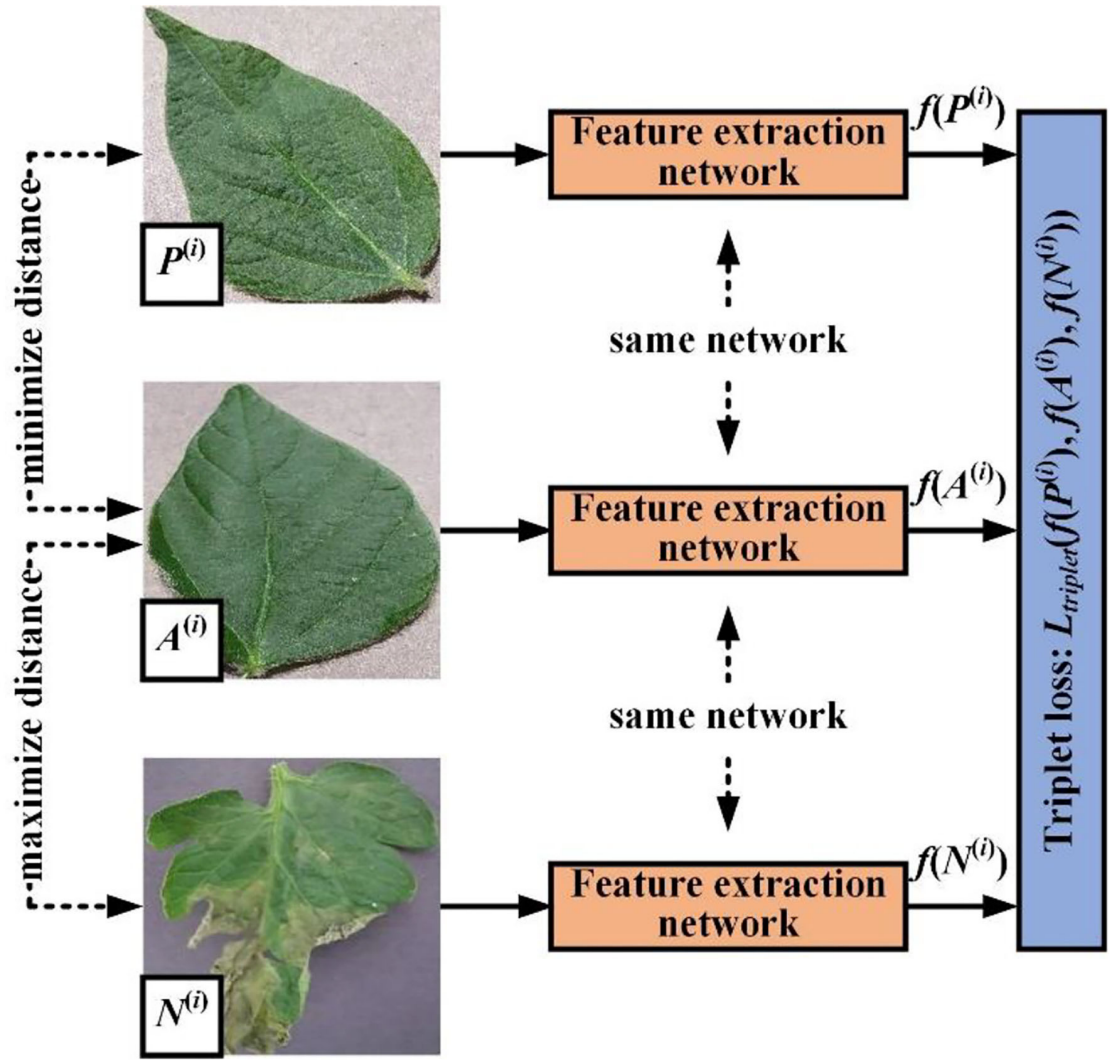
Sketch of common triplet loss calculation.

Figure 6 shows the usage of triplet loss function. The triplet loss function attempts to pull features of two images from different classes farther and push features of two images from the same class closer. Thus, our objective is:


(1) $$\begin{array}{l} \|f\left(A^{\left(i\right)}\right)-f\left(P^{\left(i\right)}\right){\|}_{2}^{2}+\alpha <\|f\left(A^{\left(i\right)}\right)-f\left(N^{\left(i\right)}\right){\|}_{2}^{2} \end{array}$$

where the function '*f* (.)' indicates our feature extraction network. *A* indicates an anchor image, *P* indicates another image from the same class, and *N* indicates an image from another different class. α is a hyperparameter called the margin that represents the minimum value between distances (*A* to *P*) and distance (*A* to *N*). In our experiment, this value is set at 0.5.

During model training, we use the Euclidean distance to minimize the triplet loss defined as:


(2) $$\begin{array}{l} L_{triplet}=\sum_{i=0}^{N}[{\left\|f\left(A^{\left(i\right)}\right)-f\left(P^{\left(i\right)}\right)\right\|}_{2}^{2}-\|f\left(A^{\left(i\right)}\right)\\ {{-f\left(N^{\left(i\right)}\right)\|}_{2}^{2}+\alpha ]}_{+} \end{array}$$

where *N* and *i* represent the number of training samples and the *i-th* training sample, respectively. The notation [*x*]+ is a hinge function (*x* stands for any function or value), representing max (*x*, 0), which can exclude the samples accurately predicted by the network from loss calculation to avoid network overfitting.

The training mode of triplet loss is divided into offline training and online training through generation rules of training samples. Offline training stands for training samples that are generated before training. However, this method is inefficient because we should perform a full forward propagation on the whole training set to generate triplets, and a lot of candidate triplets may be generated, resulting in a large batch size of each training epoch. For online training, we use image vectors of the current training batch to build useful triplets without any offline mining. Therefore, we chose the online training strategy for our task. In addition, this paper also uses the batch hard triplet loss algorithm (Hermans et al., 2017) to provide a better code implementation for online triplet mining, which can guide the model to learn a more discriminative feature vector of leaf disease.

##### Softmax loss

Label smoothing and temperature scaling in softmax loss have been certified to be useful for model training (Jun et al., 2020). Thus, we add them in our experiments to learn robust features and avoid overfitting. The softmax loss here is defined as:


(3) $$\begin{array}{l} L_{softmax}=-\frac{1}{M}\overset{M}{\underset{i=1}{\sum }}\log\frac{e^{\left(W_{yi}^{T}f\left(x_{i}\right)+b_{y_{i}}\right)/T}}{\overset{N}{\underset{j=1}{\sum }}e^{\left(W_{j}^{T}f\left(x_{i}\right)+by_{j}\right)/T}} \end{array}$$

where *M, N*, and *y*_*i*_ are the batch size, the number of classes, and class label of *i-th* input, respectively. *f* (*x*_*i*_) represents the output of the feature extractor, and *W* and *b* represent the weights and bias for the last layer of the network. *T* is a temperature parameter that can provide a softer probability distribution over classes with a higher value, which is set to 5 in our experiment. The label smoothing estimates the marginalized effect of a label dropout during training to enhance model generalization.

##### Total loss

The total loss function is defined as:


(4) $$\begin{array}{l} L_{total}=\alpha L_{triplet}+\beta L_{softmax} \end{array}$$

The hyper-parameters α and β are constant values to balance the influence of each loss term. To set the two loss functions to be at the same order of magnitude during the training process, we made α and β both equal to 1.

### Image retrieval system

**Figure 7:**
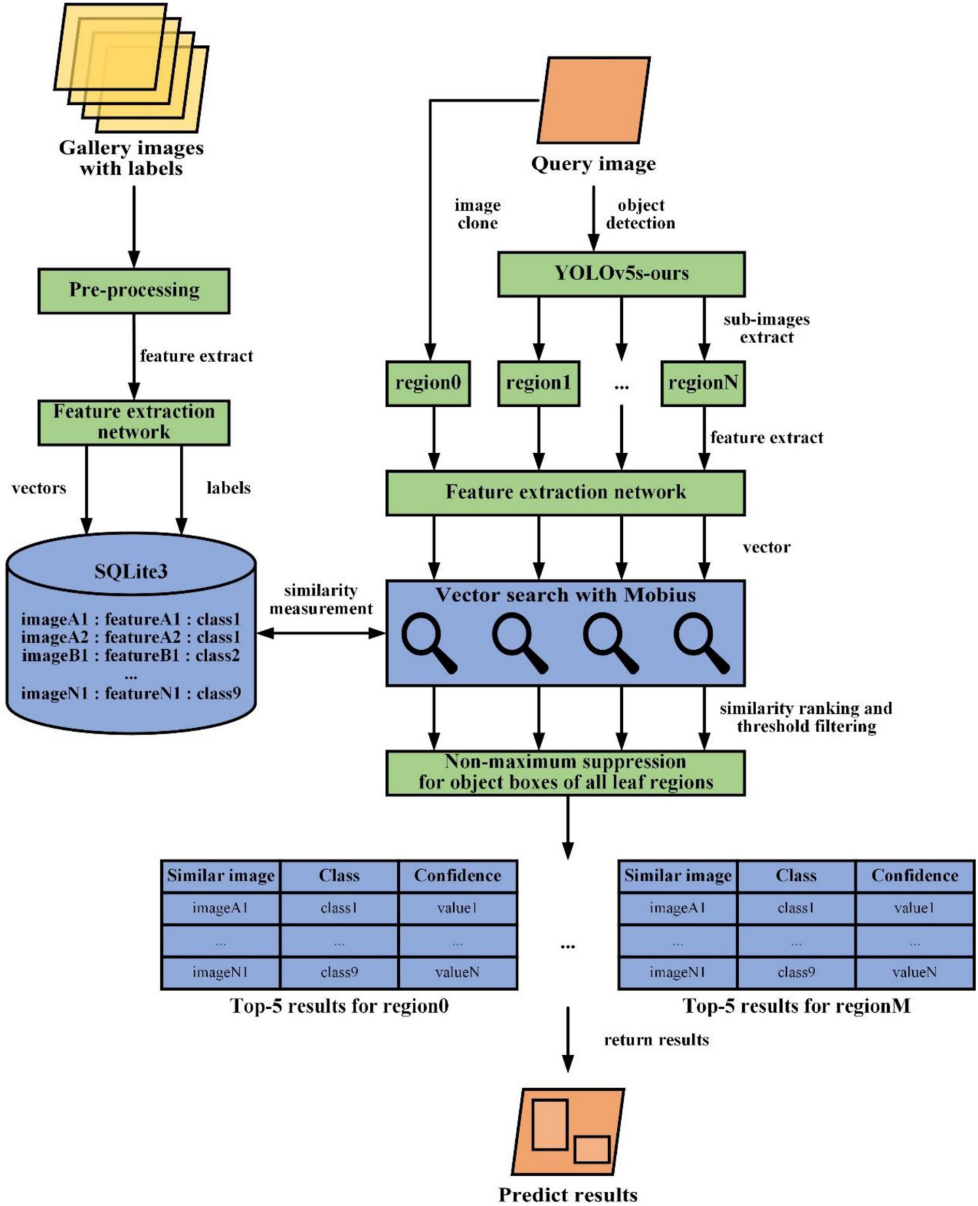
Workflow of the proposed image retrieval system.

Once the feature extraction network is trained, we can construct a vector search engine that is able to find any feature-related images from its database and return results to achieve leaf disease detection. The architecture of our vector search engine is represented in Figure 7. To find the images closest to a given query, a vector search engine needs to:

1. Calculate feature vectors of all the gallery set images through a trained feature extraction network. When we obtain a fixed-dimensional feature vector *f* (*x*) then which will be performed L2 norm normalization. The calculation formula is:
  (5) $$\begin{array}{l} \vec{F}=\frac{f\left(x\right)}{{\left\|f\left(x\right)\right\|}_{2}} \end{array}$$
  finally, we will save the feature vectors and their label information to database.
2. Get all leaf detection regions in a query image through object detection. Then, compute the feature vector of all leaf regions and the query image by a similar procedure as step 1. Adding the whole query image as a leaf region for feature extraction is to improve recall since the results of leaf object detection are not always accurate.
3. For each leaf region, similarity measurement by comparing its feature vector to all the feature vectors in our gallery dataset. Similarity is computed with a metric distance function, such as cosine distance or Euclidean distance. The smaller the Euclidean distance or the larger the cosine distance of the feature vector, the greater the feature vector similarity. Since cosine distance is easier to calculate, it is selected to represent the similarity of features. The calculation formula of cosine distance is:
  (6) $$\begin{array}{l} D_{\cos}\left({\vec{F}}_{1},{\vec{F}}_{2}\right)=\frac{{\vec{F}}_{1}\cdot {\vec{F}}_{2}}{{\left\|{\vec{F}}_{1}\right\|}_{2}{\left\|{\vec{F}}_{2}\right\|}_{2}} \end{array}$$
  where ${\vec{F}}_{1}$ and ${\vec{F}}_{2}$ represent feature vectors. Since ${\vec{F}}_{1}$ and ${\vec{F}}_{2}$ are the normalized vectors, the above equation can be simplified to:
  (7) $$\begin{array}{l} D_{\cos}\left({\vec{F}}_{1},{\vec{F}}_{2}\right)={\vec{F}}_{1}▪{\vec{F}}_{2} \end{array}$$
  However, the above implementation is an instance of a linear search with *O*(n) complexity, indicating that it will execute slowly while gallery data volume is massive. We could boost the query speed by, for example, using specialized data structures or approximate nearest neighbor algorithms that decrease the computational complexity to *O*(*logn*). Here, a simple but efficient novel graph indexing and searching algorithm will be used in our vector search task, that is Mobius (Zhou et al., 2019), a fast search on graph algorithm for maximum inner product search, which brings welcome changes in comparison with existing search mode.
4. For individual leaf regions, sort these similarity scores in descending order and filter similarity scores with confidence threshold to ensure accuracy. Then, we adopt non-maximum suppression for object boxes of all leaf regions to avoid fetching duplicate regions. Take the category of leaf image in gallery dataset with the Top-1 or Top-5 similarity scores as category prediction for the query image. In our work, we set the Top-1 score as the prediction result. At the same time, these similar images and the query image are displayed for users' reference. If all similarity scores are less than the confidence threshold, it is considered that the category of the current queried image does not appear in the gallery image.

## Results and discussion

We will give evidence that our proposed method successfully constructs a plant disease image retrieval system with remarkable inference speed and great accuracy.

### Datasets and experimental setup

#### Datasets

##### Dataset in metric learning

We evaluated our method on three typical image datasets: 1) PlantVillage dataset (Hughes and Salathe, 2016), 2) coffee leaf dataset (Esgario et al., 2020), and 3) citrus leaves dataset (Wu et al., 2007). The PlantVillage dataset was divided into a sub-dataset (PlantVillage-A) of 38,035 images and a sub-dataset (PlantVillage-B) of 16,270 images. Coffee leaf dataset (2,209 images) and citrus leaves dataset (609 images) were divided into training (coffee training, citrus training) and test set (coffee test, citrus test) with a 7:3 ratio. The training of the metric learning approach in this paper is divided into three parts.

Model training: When training a feature extraction network, it is common practice to select several categories of images in the dataset, divide these images into validation sets, and then use the remaining images as training sets. In our work, we used PlantVillage-A for training feature extraction networks. The detailed approach was that the first 19 classes were divided as the training set (PlantVillage-A-training), and the last 19 classes were divided as the validation set (PlantVillage-A-validation). PlantVillage-A-training and PlantVillage-A-validation contain 22,214 and 15,821 images, respectively.

Closed-set identification: In this instance, PlantVillage-A was used as the gallery dataset for all query images in PlantVillage-B. Through the analysis experiments on PlantVillage-A and PlantVillage-B with the same category label, the algorithm classification ability will be demonstrated.

Open-set identification: We created a gallery set from PlantVillage-A, coffee training, citrus training, and a query set from coffee test, citrus test. Since most of the classes in coffee dataset and citrus dataset did not appear in Plantvillage-A, it would highly evaluate the generalization ability of our image retrieval system.

##### Dataset in object detection

In our work, the experimental dataset for leaf detection used public datasets supplied by Flavia (Wu et al., 2007), Swedish (Söderkvist, 2001), Leafsnap (Kumar et al., 2012). Flavia consists of 1,907 leaf images divided into 32 categories, which were sampled in Nanjing, China. Swedish contains 15 different Swedish tree species, with 75 images per species for a total of 1,125 images. Leafsnap consists of 7,719 images (800 × 600 pixels), covering all 185 tree species from the Northeastern United States. Moreover, we used web crawlers to capture 1,500 leaf images and introduced these images and PlantVillage-A (38,035 images) into experiments. PlantVillage-A dataset and Leafsnap dataset provide leaf location segmentation information that can be used to generate object bounding boxes. For other datasets, we labeled bounding boxes for each image manually. The above images were then cleaned and mixed to create a leaf location dataset containing 50,000 images. Our object detection model was trained to only perform one class object detection, namely, a leaf object.

#### Experimental setup

Hardware Platforms: All experimental codes were executed on Python 3.7.10 with Pytorch1.8 and CUDA 10. We used a cloud server with two Intel Xeon CPUs, eight NVIDIA Tesla V100 GPUs, and 256 GB memory to train models. Model evaluation was conducted on a local server with an Intel Core CPU, an NVIDIA GTX 1060ti GPU, and 16 GB memory.

Model training: For object detection tasks, all models used in this study are initialized by the COCO pretrained YOLOv5 series models. Furthermore, multi-scale training and additional enhancement testing techniques were not used in these tasks. For metric learning tasks, we used the ImageNet pretrained model to initialize the backbone network. When training models, we used the Tree-structured Parzen Estimator (TPE) approach (Bergstra et al., 2011) for tuning the hyperparameters to obtain the best model. All networks were fixed and trained for 100 epochs using the stochastic gradient decent optimization algorithm. Then, defined three hyperparameters, that is, batch size, initial learning rate, and weight decay, will be chosen by TPE to optimize performance. For each model training, the total number of parameter search trials in TPE was 20. The early stopping mechanism was configured in TPE to speed up model training, which was set to stop the current search trial if any ten consecutive epochs with no advancement in reducing training loss.

### Leaf detection algorithm

The YOLOv5-ours and different YOLOv5 architectures, and whose different resolutions input size, were tested and evaluated for the leaf object detection task. We will use mean average precision (mAP) as the selected metric to quantitatively compare their performance. The evaluation in this paper was based on mAP@0.5, which was used as comprehensive evaluation metrics, where mAP@0.5 was the mAP calculated under the intersection over union (IOU) threshold of 0.5 (Song et al., 2021). Quantitative comparisons of the proposed improved detection method against YOLOv5s and different structure models of YOLOv5 are shown in Table 1, which give a comparison of different networks with different dimensions of feature vectors in mAP@0.5 (accuracy), inference time (speed), and model size (the number of model parameters).

**Table 1:** Performance on leaf location testing set for each object detection method.

**Methods**	**Input size**	**mAP@0.5** **(%)**	**Inference** **time (ms)**	**Model** **size (MB)**
YOLOv5-ours	320X320	98.03	4.52	14.24
	416X416	98.17	6.33	
	608X608	98.00	13.11	
YOLOv5s	320X320	97.76	3.31	13.69
	416X416	97.96	4.73	
	608X608	97.87	10.14	
YOLOv5m	320X320	97.86	7.75	40.47
	416X416	98.12	12.16	
	608X608	97.94	28.87	
YOLOv5l	320X320	98.02	13.18	89.35
	416X416	98.22	20.29	
	608X608	98.06	48.60	
YOLOv5x	320X320	98.23	23.61	166.93
	416X416	98.43	36.42	
	608X608	98.33	95.31	

YOLOv5-ours has better test accuracy than YOLOv5s and YOLOv5m, and comparable performance with YOLOv5l and YOLOv5x. Obviously, mAP value of YOLOv5s was the lowest, but inference speed and model size were the fastest. YOLOv5x has the best detection accuracy but demands more inference time than other detection models. Compared with YOLOv5s, the detection accuracy of our model was substantially improved at the expense of a tiny amount of inference speed and model size.

Model complexity introduced by BiFPN structure and added a small object detection layer only increases a small amount of model storage space and inference time in our proposed model, and overall capability has clear superiority compared with heavy-weight networks. The comparison results in different model input sizes, particularly under 320 input sizes, exhibit the power of our model in improving the accuracy of small object detection. This result demonstrates that our proposed approach has a good trade-off between accuracy and computational efficiency. An input size of 416 × 416 was simpler to obtain wonderful results than others on most occasions. So, we used input size of 416 × 416 as the input image size of our detection network in subsequent tasks.

### Analysis of leaf disease recognition algorithms

In this part, we showed the performance of our feature networks on PlantVillage-A datasets. After that, we explored the contributions of each proposed module by ablation experiments.

#### Experimental results on PlantVillage-A

Top-k accuracy was a widespread evaluation standard, which means that the model outputs the most probable k classification results, and the output is regarded as correct when those k results contain the actual class label. We used Top-1 test accuracy for feature network model evaluation during the training stage. Table 2 lists the performance of our networks as compared to different dimensions of feature vectors.

**Table 2:** Performance for feature extraction networks with different dimensions of feature vectors.

**Backbone**	**Dimension** **of feature** **vectors**	**Top-1 (%)**	**Inference** **time (ms)**	**Model** **size (MB)**
ResNet-50	128	97.22	14.07	90.92
ResNet-50	256	97.39	14.15	91.94
ResNet-50	512	**97.88**	14.22	93.98

The feature extraction networks built on the backbone network being ResNet50 with an output feature vector of 512 dimensions achieved the highest performance with a validation accuracy of 97.88%. However, networks with different output dimensions have all shown good performance in this context, which indicated a sure sign that we were on the right track to achieve higher accuracy of leaf disease identification. Obviously, a larger output feature dimension improved identification performance, yet increased model computing and storage resources consumption. From our experimental results, the 512 dimensions of output did not bring excessive resource consumption relative to others. So, we considered that was acceptable and set the output feature vector of all feature extraction networks to 512 dimensions in subsequent image retrieval stages.

#### The impact of backbone

To further explore the potential performance improvement on different backbone networks for leaf disease recognition, we performed ablation analysis on the PlantVillage-A dataset. A variety of classification networks were used to build feature extraction networks based on metric learning, and the dimension of the feature vector was adjusted to 512.

Compared with ResNet50 as the backbone network, ResNet101 and ResNet152 increased validation accuracy by 0.14 and 0.65%, respectively (Table 3). When the feature vector length was 512, the feature extraction network based on ResNet152 achieved the highest recognition accuracy of 98.53% (Table 3). Yet it is worth noting that the inference time and model size for both ResNet101 and ResNet152 were much larger than that in ResNet50. Therefore, in actual application, it is possible to choose a proper backbone network according to the requirements of system performance and recognition accuracy. Inference times of transformers and MobileNet vary too much depending on physical hardware architecture. These data are not listed in our paper because they were not representative and intangible on our platform.

**Table 3:** Comparisons of different backbone networks on validation sets.

**Backbone**	**Top-1 (%)**	**Inference** **time (ms)**	**Model** **size (MB)**
MobileNetV3-Large	97.21	-	18.72
ResNet101	98.02	28.31	166.65
ResNet152	98.53	40.79	226.53
ResNet50	97.88	14.22	93.98
EfficientNet-B0	98.01	20.63	18.06
Vit-Base-patch16	98.73	-	328.89
Deit-Base-patch16	98.92	-	328.89

However, no matter which common backbone network we chose, our algorithm still achieved good performance. In short, our results showed that choosing ResNet50 as the backbone network, albeit not optimal in accuracy, has the highest performance price ratio, which can acquire trade-offs between time and accuracy, and it was the best solution for our task.

#### The impact of model loss algorithms

We designed a controlled experiment to explore the contribution of classification loss and triple loss to the feature extraction network. The detailed experimental results are shown in Table 4.

**Table 4:** Comparisons of different loss functions on validation sets.

**Backbone**	**Loss**	**Top-1 (%)**
ResNet50	Tripletloss + softmax loss	97.88
ResNet50	Triplet loss	97.45
ResNet50	Softmax loss	96.93

The feature extraction network training with softmax loss and triplet loss was the baseline model. Here, we discussed model accuracy after removing triplet loss and softmax loss in the baseline model, respectively. Compared with the baseline model, recognition accuracy of the model trained with softmax loss was reduced by 0.95%, while that trained by triplet loss was only reduced by 0.43%. With or without classification loss, our model maintains satisfactory recognition accuracy. However, compared with using only triple loss, adopting the joint learning strategy combining softmax loss and triplet loss can bring high accuracy gain.

### Closed-set identification and open-set identification

#### Experimental results of recognition system

The main aim of our image retrieval system was to efficiently find relevant images from a dataset given a query image, thereby determining the category of the query image. To judge how our image retrieval system performance, Top-1 and Top-5 accuracy were employed as the evaluation metric for all test datasets. Table 5 exhibits Top-1 accuracy, Top-5 accuracy, and inference time of our image retrieval system on the test set.

**Table 5:** Performance of our image retrieval system on different test sets.

**Datasets**	**Backbone**	**Top-1** **(%)**	**Top-5** **(%)**	**Inference** **time** **(ms)**	**Identification** **mode**
PlantVillage-B	ResNet50	97.84	99.52	32.48	Close-set identification
Coffee test	ResNet50	89.09	99.09	33.36	Open-set identification
Citrus test	ResNet50	91.67	98.89	32.65	Open-set identification

For close-set identification, our image retrieval system obtained 97.84 and 99.52% Top-1 and Top-5 classification accuracy, respectively, while it takes 32.48 ms to accomplish per detection on average. The computation time of recognition process includes leaf detection time, feature extraction time, and image matching time. These results showed that our system can recognize leaf disease on the PlantVillage-B set well.

We also explored the generalization capability of our image retrieval system in unknown datasets (open-set), those were coffee test and citrus test. As shown in Table 5, for test results of coffee test and citrus test, Top-1 accuracy rates were 89.09 and 91.67%, and Top-5 accuracy rates were 99.09 and 98.89%, respectively. The feature search was carried out on the GPU mode with C/C++ implementation. Our system achieved a faster search speed, and the running time for the test on both datasets was less than 34 ms.

**Figure 8:**
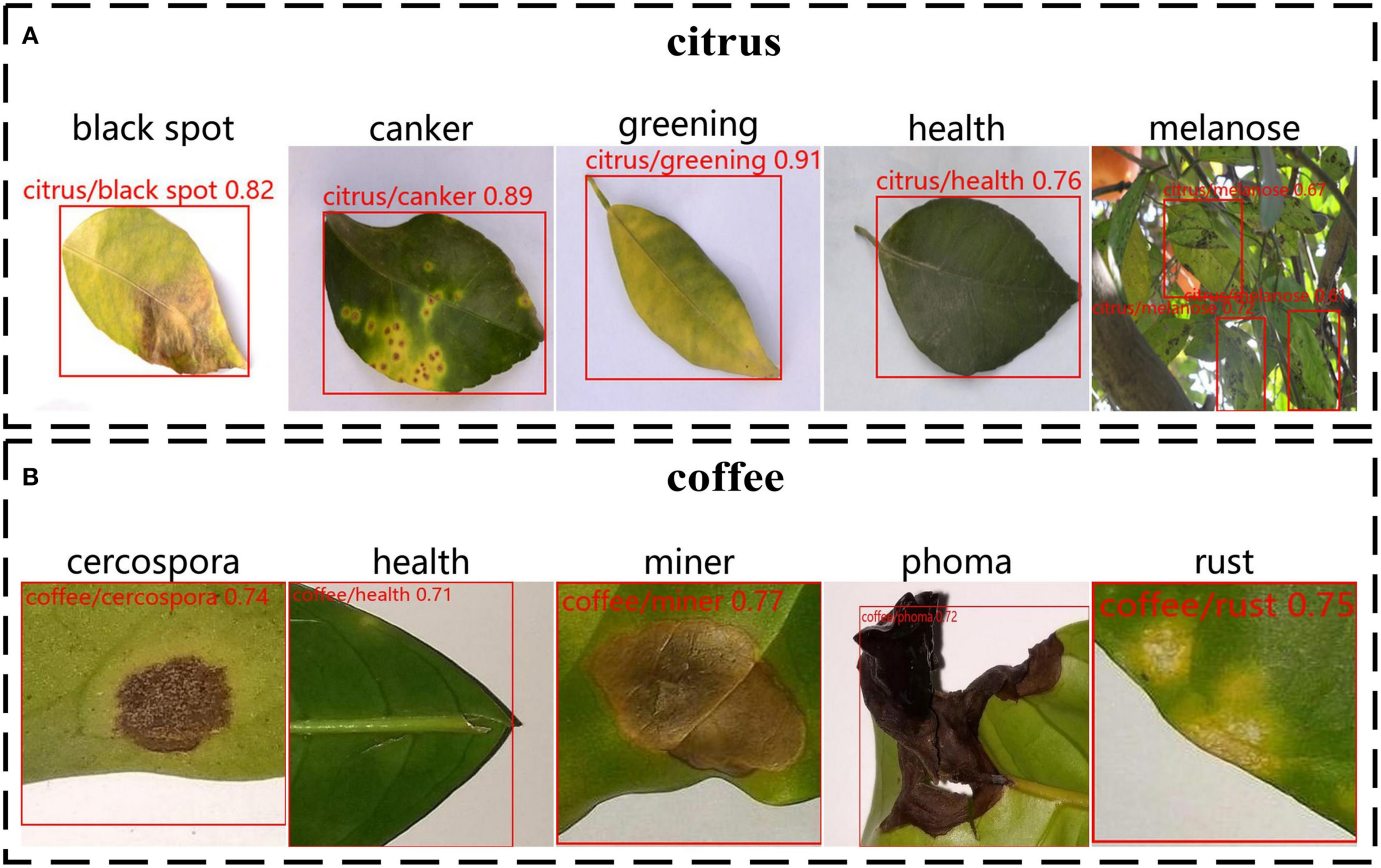
An identification example of open-set identification on citrus datasets and coffee datasets. The red zone in the diagram indicates a leaf location. The description text on each image indicates the type of disease and the confidence of recognition. **(A)** Recognition results of diseases on citrus leaf images. **(B)** Recognition results of diseases on coffee leaf images.

An identification example of open-set identification is shown in Figure 8. Using our image retrieval system, we obtained the following two results. Figure 8A is the test results in citrus test; Figure 8B is the test results in the coffee test. Our improved YOLOv5 can accurately locate leaf objects, especially small leaf objects. The proposed retrieval system demonstrated good performance and successfully gave accurate identification results and confidence for query images with different types of leaf diseases. Although new plant disease types in the test set are not present in the training set, our image retrieval system was able to recognize new classes when test images were loaded in the retrieval system, which indicates that the features extracted by the feature extraction network have sufficient discrimination capability and proves the effectiveness of metric learning.

#### Robustness analysis of recognition system

**Figure 9:**
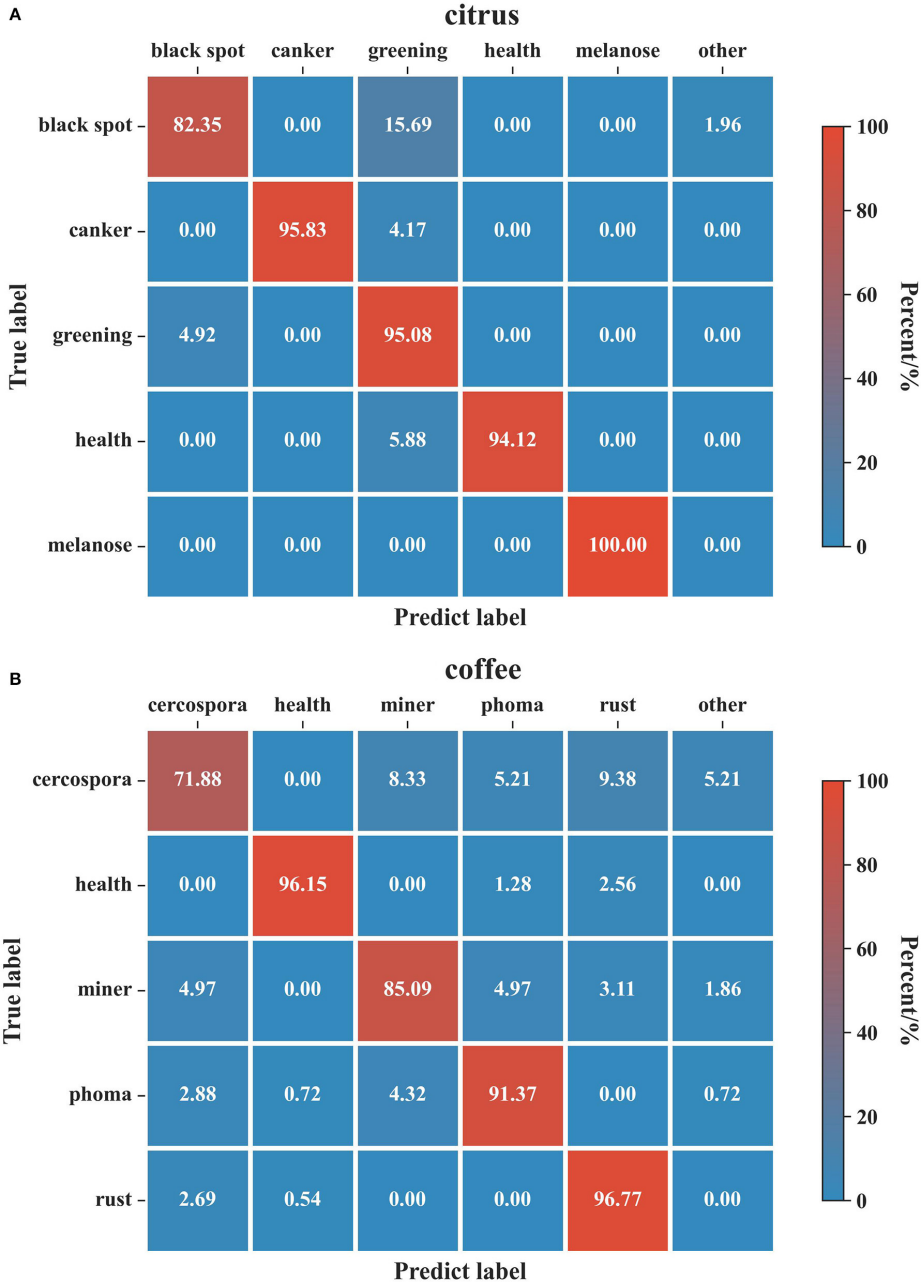
Confusion matrix for the evaluation results on the citrus and coffee datasets. **(A)** Citrus dataset. **(B)** Coffee dataset. The columns of the confusion matrix indicate the predicted classes and the rows correspond to the true classes. The diagonal represents the ratio of true positives whereas the rest of the matrix corresponds to false negatives. A detection result whose probability is less than the threshold is set as the class: ‘other'.

There is no doubt that conducting analysis experiments on the more challenging open-set recognition task can demonstrate the model robustness. To this end, the classification confusion matrixes on the two datasets of open-set recognition tasks are plotted in Figure 9.

In Figure 9, the columns of the confusion matrix indicate the predicted classes, and the rows correspond to the true classes. The diagonal represents the ratio of true positives, whereas the rest of the matrix corresponds to false negatives. A detection result whose probability is less than the threshold is set as the class: “other”. As can be seen from the results of citrus, our models performed well in “canker”, “black spot”, “healthy”, and “melanose” classes, achieving greater than 94% classification accuracy. However, it can be observed that the lowest accuracy is reported for the “black spot” disease since some of the samples from the “black spot” have evidence of “greening” disease. Not surprisingly, the major portion of the failed samples from the “greening” class was also classified as “black spot”. This behavior is because the black spot symptoms tend to blend with the “greening” disease symptoms when the severity of the “black spot” disease is not intense. Likewise, for coffee datasets, the results of the most disease classes have good accuracy except for “cercospora” disease which displayed a considerable number of classification errors. This result is consistent with the experiments carried out in coffee leaf by Esgario et al. (2020) whose class with the largest number of samples misclassified was also the “cercospora” disease.

We can alleviate these problems by improving the input resolution of the model when the hardware conditions allow. Further, to go for a higher accuracy model, one can use a heavier backbone model or higher feature dimensions.

#### Comparison with classification results of previous studies

The experimental results of our image retrieval system obtained are not directly comparable with classification methods in the previous. However, there is some consistency concerning the results obtained with those of classification methods. For fair comparison, we compare the accuracy of our method and different classification models on citrus and coffee datasets.

For the citrus datasets, Janarthan et al. (2020) proposed a patch-based classification network that comprises an embedding module, a cluster prototype module, and a simple neural network classifier, to reach the accuracy rate of 95.04%. Syed-Ab-Rahman et al. (2022) employed a two-stage deep CNN model for citrus disease classification using leaf images, whose model delivers 94.37% accuracy in detection. For the coffee datasets, Esgario et al. (2020) proposed a multi-task system based on convolutional neural networks and achieved 97.07% accuracy.

From the above results, the existing deep learning-based methods usually employ classification models to achieve detection of plant diseases. These methods all aim at specific datasets via the data-driven manner to build classification models, which can achieve high classification performance. In contrast, our model achieved 91.67 and 89.09% accuracy in citrus and coffee datasets. Compared with image classification methods, image retrieval has a gap in recognition accuracy. But our image retrieval system offers great versatility and tunability characteristics, which means we can obtain sufficient recognition accuracy in unknown class datasets without rebuilding and retraining the model. For the environment where plants suffer from many kinds of diseases and insect pests, our image retrieval system can be quickly applied at minimal implementation cost.

### Discussion on algorithm applications

The extensive experimental results demonstrate the feasibility and validity of our proposed image retrieval system in leaf disease recognition. However, the recognition system shall be adjusted according to its actual application situation.

In our work, leaf detection largely dictates the accuracy of subsequent plant disease retrieval. In the experiments, we locate leaves through the improved YOlOv5s. In a complex dynamic environment, a larger detection model or higher image resolution is needed to maintain detection accuracy but this change is at the expense of detection speed and application performance. Therefore, to make a trade-off between accuracy and speed according to the actual situation, the model can be set by referring to the results in Table 2.

If users need to apply our system to edge devices with weak hardware performance, they should do some extra work on the system to speed up model inference. Firstly, some model compression techniques (Chen et al., 2020), including pruning, quantification, and model distillation, can be used to compress leaf detection models and leaf disease retrieval models. Secondly, according to our experimental results, smaller backbone models and smaller feature sizes can speed up the inference of image retrieval model, but only with a small loss of accuracy. Finally, using a universal and efficient inference engine is a more efficient and common acceleration method for model deployment (Jiang et al., 2020). If the edge device can perform network communication, the recognition system can be deployed on a high-performance remote server, and the edge device only needs to send images and display results.

Use different levels to indicate the severity range recognition of a plant disease, and representing each level as a category. Like this, we can measure the severity of the symptoms on the target leaf. Further, we can train our detection model to detect new categories of objects, such as the different organs of plants. Then, collect images of different disease categories of other plant organs and retrain the recognition model. In this way, our detection system can be extended to the disease recognition of different plant organs.

## Conclusions

This study focuses on a more common and challenging scenario, namely, the open-set identification of leaf disease. With regard to this, we proposed a new image retrieval system that simultaneously produces leaf diseases localization and identification with limited annotation images. This opens up the probability of our model being able to accurately identify leaf disease even though it has encountered a new type never before seen by the model. For the task of detecting leaf objects, we have improved YOLOv5s, which has higher overall accuracy and performs better in small object detection. We believe that CNNs built with metric learning are more suitable for our retrieval tasks. These methods leave a lot of room for improvement since they fail to take advantage of class labels. Toward this end, we combine metric learning with classification prediction, empowering our networks to make full use of the classification capability of CNNs and acquire great recognition performance. We also chose ResNet50 as the backbone network to extract features at various levels, which balances efficiency and accuracy. We ultimately employ Mobius for fast vector search and integrate various algorithm modules to build a retrieval system to find out matching images for a given image.

The extensive experimental results prove the feasibility and validity of our proposed image retrieval system in leaf disease recognition. With a new leaf disease category, we only need to put images of that category into the retrieval library; thereby, our retrieval system can identify this new leaf disease without further retraining. Future work focuses on ensemble vision transformer and CNN for image retrieval and pursuing better overall performance.

## Data availability statement

The original contributions presented in the study are included in the article/supplementary materials, further inquiries can be directed to the corresponding author/s.

## Author contributions

YP: conceptualization, methodology, resources, software, validation, and writing-original draft. YW: visualization, software, and writing-review and editing. All authors contributed to the article and approved the submitted version.

## Funding

This work was supported by the Doctorate Fellowship Foundation of Nanjing Forestry University (163010550), the Priority Academic Program Development of Jiangsu High Education Institutions (PAPD), and Special Project of ‘Lushan Plants' (2022ZWZX02).

## Conflict of interest

Author YW was employed by Jiangsu Wiscom Technology Co. Ltd. The remaining author declares that the research was conducted in the absence of any commercial or financial relationships that could be construed as a potential conflict of interest.

## Publisher's note

All claims expressed in this article are solely those of the authors and do not necessarily represent those of their affiliated organizations, or those of the publisher, the editors and the reviewers. Any product that may be evaluated in this article, or claim that may be made by its manufacturer, is not guaranteed or endorsed by the publisher.
